# Vitamin B6 deficiency cooperates with oncogenic *Ras* to induce malignant tumors in *Drosophila*

**DOI:** 10.1038/s41419-024-06787-3

**Published:** 2024-06-03

**Authors:** Eleonora Pilesi, Giulia Tesoriere, Angelo Ferriero, Elisa Mascolo, Francesco Liguori, Luca Argirò, Chiara Angioli, Angela Tramonti, Roberto Contestabile, Cinzia Volontè, Fiammetta Vernì

**Affiliations:** 1 Dept. of Biology and Biotechnology “Charles Darwin”, Sapienza University of Rome, 00185 Rome, Italy; 2 Experimental Neuroscience and Neurological Disease Models, IRCCS Santa Lucia Foundation, 00143 Rome, Italy; 3 CNR, Institute for Systems Analysis and Computer Science, 00185 Rome, Italy; 4 Institute of Molecular Biology and Pathology, 00185 Rome, Italy; 5 Department of Biochemical Sciences “A. Rossi Fanelli”, Sapienza, University of Rome, 00185 Rome, Italy; 6 Istituto Pasteur-Fondazione Cenci Bolognetti, Sapienza, University of Rome, 00185 Rome, Italy

**Keywords:** Cancer metabolism, Diseases

## Abstract

Vitamin B6 is a water-soluble vitamin which possesses antioxidant properties. Its catalytically active form, pyridoxal 5’-phosphate (PLP), is a crucial cofactor for DNA and amino acid metabolism. The inverse correlation between vitamin B6 and cancer risk has been observed in several studies, although dietary vitamin B6 intake sometimes failed to confirm this association. However, the molecular link between vitamin B6 and cancer remains elusive. Previous work has shown that vitamin B6 deficiency causes chromosome aberrations (CABs) in *Drosophila* and human cells, suggesting that genome instability may correlate the lack of this vitamin to cancer. Here we provide evidence in support of this hypothesis. Firstly, we show that PLP deficiency, induced by the PLP antagonists 4-deoxypyridoxine (4DP) or ginkgotoxin (GT), promoted tumorigenesis in eye larval discs transforming benign *Ras*^*V12*^ tumors into aggressive forms. In contrast, PLP supplementation reduced the development of tumors. We also show that low PLP levels, induced by 4DP or by silencing the *sgll*^*PNPO*^ gene involved in PLP biosynthesis, worsened the tumor phenotype in another *Drosophila* cancer model generated by concomitantly activating Ras^V12^ and downregulating *Discs-large (Dlg)* gene. Moreover, we found that *Ras*^*V12*^ eye discs from larvae reared on 4DP displayed CABs, reactive oxygen species (ROS) and low catalytic activity of serine hydroxymethyltransferase (SHMT), a PLP-dependent enzyme involved in thymidylate (dTMP) biosynthesis, in turn required for DNA replication and repair. Feeding *Ras*^*V12*^ 4DP-fed larvae with PLP or ascorbic acid (AA) plus dTMP, rescued both CABs and tumors. The same effect was produced by overexpressing catalase in *Ras*^*V12*^ *Dlg*^*RNAi*^ 4DP-fed larvae, thus allowing to establish a relationship between PLP deficiency, CABs, and cancer. Overall, our data provide the first in vivo demonstration that PLP deficiency can impact on cancer by increasing genome instability, which is in turn mediated by ROS and reduced dTMP levels.

## Introduction

The catalytically active form of vitamin B6, pyridoxal 5′-phosphate (PLP), is an essential cofactor for a wide range of metabolic reactions mostly regulating amino acid biosynthesis and catabolism [1, 2]; in addition, PLP participates, as a cofactor of serine hydroxymethyltransferase (SHMT), to one-carbon (1 C) metabolism, involved in DNA synthesis and methylation processes [3]. Furthermore, vitamin B6 can quench reactive oxygen species (ROS) [4–6] and it regulates both abiotic and biotic stress of plants [7]. In animals, PLP is recycled from food in a salvage pathway requiring the action of pyridoxal kinase (PDXK) and pyridoxine 5’-phosphate oxidase (PNPO) enzymes [8]. Vitamin B6 is present in most foods, therefore a primary deficiency is uncommon in developed countries. However, a deficiency of vitamin B6 can develop as a secondary effect of several common pathologies including diabetes, celiac disease and bowel syndrome [8]. In addition, PLP deficiency is a condition often associated with pregnancy and can originate as a side effect of many common drugs including antibiotics such as isoniazid, penicillamine and cycloserine [9].

Vitamin B6 has been inversely associated to the risk and/or survival of several cancers [10]. Reduced serum levels of PLP, as well as impaired functional vitamin B6 status, as evaluated by the 3-hydroxykynurenine: xanthurenic acid ratio (HK:XA), have been for instance related to lung cancer risk [11–13]. Consistently, high expression levels of *PDXK* have been positively correlated to survival of non-small cell lung cancer (NSCLC) patients [14]. Low levels of plasmatic PLP have been related also to colon-rectal cancer (CRC) risk in several studies [15–19], while high levels have been associated with improved survival among CRC patients [20].

In contrast, previous observational studies of dietary or dietary plus supplementary intake of vitamin B6 and CRC risk reported nonsignificant positive or inverse associations [21, 22]. However a recent meta-analysis [23] including new studies [24, 25] resolved this discrepancy.

Works performed in mice and rats have shown that dietary vitamin B6 suppresses colon [26] and mammary tumorigenesis [27], thus indicating that animal models may help overcome limits and bias often associated to human research and, more importantly, can give the possibility to elucidate molecular links.

Based on multiple roles of vitamin B6, several mechanisms have been proposed to explain its impact on cancer. In particular, it is thought that PLP deficiency may compromise DNA synthesis [28], alter methylation pattern [29], promote angiogenesis [30], increase oxidative stress [31], promote inflammation [32] and impair anti-tumor immunity [33]. However, the molecular basis of these mechanisms has yet to be elucidated.

We and other authors have shown that PLP deficiency causes chromosome aberrations (CABs) in yeast, *Drosophila* and human cells [34, 35]. In particular, we proved that CABs originate from both oxidative stress [35] and impaired SHMT activity which in turn reduces thymidylate (dTMP) biosynthesis [36]. This finding led us to speculate that genome instability may link vitamin B6 with tumors. Consistent with this hypothesis, we also found that two PLP antagonists, 4-deoxypyridoxine (4DP) and ginkgotoxin (GT), induce the development of epithelial cancers on the adult cuticle of flies by promoting the loss of heterozygosity at the tumor suppressor *warts (wts)* locus [37].

In this work we used *Drosophila melanogaster* to investigate the possible role of vitamin B6 deficiency in cancer. *Drosophila* is emerging as a valuable model to study multiple aspects of tumor formation and malignant transformations, due to the conservation of most signaling pathways involved in cancer; in addition, flies are easy to manipulate genetically and have limited genetic redundancy. Most cancer research in *Drosophila* relied on the MARCM (mosaic analysis with repressible cell marker) strategy [38]. This allows, by mitotic recombination, the generation of clones expressing a constitutively active form of Ras85D (Ras^V12^) that are, at the same time, mutants for tumor suppressors and marked with the green florescent protein (GFP), for easy visualization. The *Ras*^*V12*^ overexpression leads to benign hyperplastic growth [39] thus, seminal works using MARCM method identified mutations in genes involved in apical–basal polarity and in genes with mitochondrial functions or involved in autophagy that cooperate with *Ras*^*V12*^ to promote malignant traits [40–43]. In addition, several studies have successfully generated *Drosophila* models for lung, colorectal, brain and thyroid cancer, and for cancers that have been proven amenable to pharmacologic approaches [44–46].

In the present work we exploited the advantages of using flies to study the possible role of vitamin B6 deficiency in tumor onset or development and to identify molecular links mainly correlated to genome stability.

## Results

### Effect of PLP deficiency on Ras^V12^*Drosophila* cancer model

We previously demonstrated that *Drosophila melanogaster* is a suitable model to study the impact of vitamin B6 on human health [35, 47]. Here, we investigated the possible role of vitamin B6 deficiency in transforming benign *Ras*^*V12*^ in malignant forms of tumor, based on the notion that cancer is a multistep process [48]. Using the MARCM strategy [38] we generated larvae (hereinafter named *Ras*^*V12*^ larvae) in which clones expressing both *Ras*^*V12*^ and the green fluorescent protein (GFP) were produced on eye-antennal discs by an *eyeless*-specific flippase enzyme (ey-FLP) via somatic recombination.

**Fig. 1:**
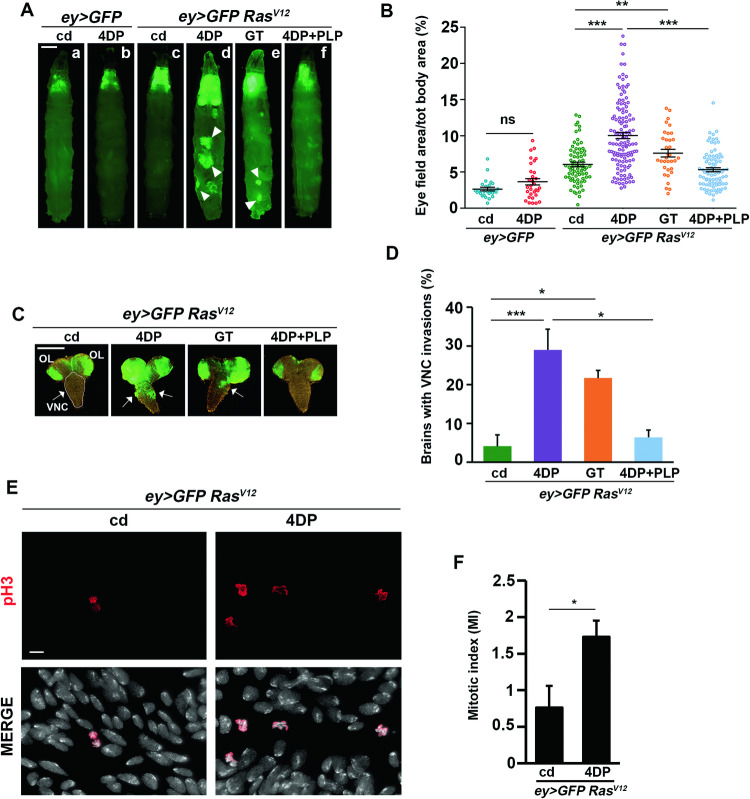
PLP deficiency cooperates with *Ras*^*V12*^ in tumorigenesis. **A** *ey* > *GFP Ras*^*V12*^ and *ey* > *GFP* control larvae reared on cd (a,c), 4DP (b,d) GT (e) or 4DP + PLP (f). *ey* > *GFP Ras*^*V12*^ larvae express both the oncogenic *Ras*^*V12*^ and the GFP protein in eye disc clones; control larvae express only the GFP protein in eye disc clones. Ectopic expression of *Ras*^*V12*^ alone only induces mild tumor growth (c). *Ras*^*V12*^ 4DP and *Ras*^*V12*^ GT tumors display significant overgrowth (d,e), which can be rescued by PLP supplementation (f). Secondary tumors far from cephalic area are indicated by arrows. cd=control diet; 4DP = 4-deoxypyridoxine; GT=gingkotoxin; PLP=pyridoxal 5’-phosphate. Scale bar, 0.5 mm. **B** Quantification of GFP-positive eye field area relative to total body area. Error bars, SEM. **P < 0.01, ***P < 0.001, ns= not significant (P = 0.0605) (unpaired *t*-test). *ey* > *GFP* cd *n* = 26; *ey* > *GFP* 4DP *n* = 31; *ey* > *GFP Ras*^*V12*^ cd *n* = 73; *ey* > *GFP Ras*^*V12*^ 4DP *n* = 129; *ey* > *GFP Ras*^*V12*^ GT *n* = 34; *ey* > *GFP Ras*^*V12*^ 4DP + PLP *n* = 82. **C** larval brains showing the invasion of GFP-labeled cells into the ventral nerve cord (VNC), arrowed. Note that GFP, in addition to eye discs, is also expressed in the optic lobes (OL). Scale bar, 100 µm. **D** Quantification of VNC invasions. Error bars, SEM. *P < 0.05, ***P < 0.001 (chi square test) *ey* > *GFP Ras*^*V12*^ cd *n* = 48; *ey* > *GFP Ras*^*V12*^ 4DP *n* = 55; *ey* > *GFP Ras*^*V12*^ GT *n* = 46; *ey* > *GFP Ras*^*V12*^ 4DP + PLP *n* = 31). **E** Examples of eye disc cells from *ey* > *GFP Ras*^*V12*^ and *ey* > *GFP Ras*^*V12*^ 4DP larvae stained with an anti-pH3 antibody to evaluate the mitotic index (MI). Lower panels show merged images for DAPI and pH3. Scale bar, 5 µm. **F** MI is expressed as the percentage of cells in mitosis. Error bars, SEM. *P < 0.05 (unpaired *t*-test). No. of examined cells: *ey* > *GFP Ras*^*V12*^ *n* = 13130 (5 discs); *ey* > *GFP Ras*^*V12*^ 4DP *n* = 19381 (3 discs).

PLP deficiency was induced by feeding *Ras*^*V12*^ larvae with the PLP antagonist 4-deoxypyridoxine (4DP [49]). As a control, in addition to *Ras*^*V12*^ larvae reared on a standard medium, we generated larvae expressing only the GFP protein in eye disc clones, to monitor possible effects of 4DP on GFP expression. 4DP feeding leads to a 3-day delay in completion of larval development [47]. Thus, third instar *Ras*^*V12*^ 4DP-fed larvae were examined at 11 days after egg laying (AEL) and compared to *Ras*^*V12*^ larvae at 8 days AEL. As reported in Fig. S1 older larval age of *Ras*^*V12*^ 4DP larvae did not influence the phenotype. 4DP feeding did not significantly alter GFP expression in control larvae (Fig. 1A, B). In contrast, it stimulated the proliferation of eye disc clones in *Ras*^*V12*^ larvae, thus increasing the percentage of GFP-positive eye field area/ total body area with respect to control diet (10% vs 6%) (Fig. 1A, B). Accordingly, eye disc cells from *Ras*^*V12*^ 4DP larvae stained for phospho-Histone H3 (pH3), a specific marker for mitotic cells, displayed an increased mitotic index (MI) (Fig. 1E, F).

Feeding *Ras*^*V12*^ larvae with gingkotoxin (GT), another PLP antagonist of PLP synthesis [49], yielded the same results (Fig. 1A, B), thus confirming the specificity of the effect. In contrast, PLP administration prevented tumor development (Fig. 1A, B), further reinforcing the role of PLP deficiency in *Ras*^*V12*^ tumor transformation.

*Ras*^*V12*^ larvae treated with PLP antagonists displayed secondary tumors far from the cephalic region (Fig. 1A). To quantify them, we analyzed the invasions on the larval brain ventral nerve cord (VNC) according to [41] and found that 4DP feeding caused VNC invasions in 29% of examined brains vs 4.1% in controls. GT treatment yielded VNC invasions (21.7%), whereas, in contrast, PLP supplementation strongly reduced migration (6.4%) (Fig. 1C, D).

We also tested 4DP on a cancer model in which *Ras*^*V12*^ was expressed in all cells of the eye disc, finding the same results (Fig. S2).

**Fig. 2:**
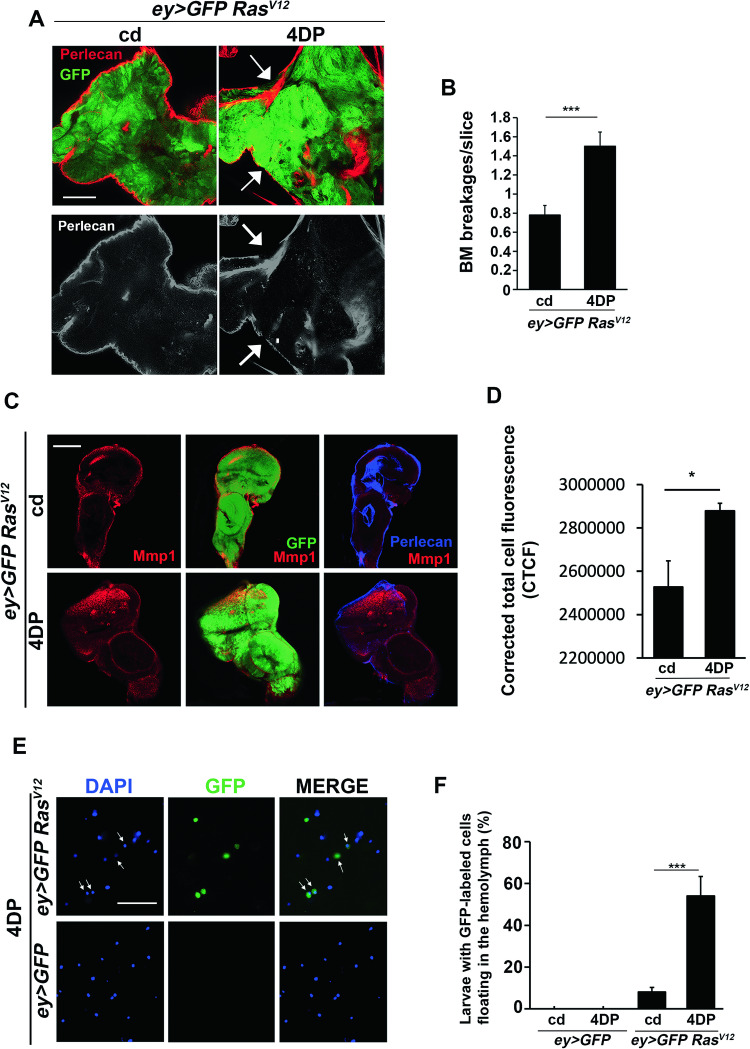
PLP deficiency causes basement membrane degradation and promotes GFP-cells migration through hemolymph. **A** Immunofluorescence on eye imaginal discs from *ey* > *GFP Ras*^*V12*^ and *ey* > *GFP Ras*^*V12*^ 4DP third instar larvae using an anti-Perlecan antibody against a core component of the basement membrane (BM). Panels are single slices obtained through Z-stack confocal acquisition. Arrows indicate BM damage. Scale bar, 20 µm. cd=control diet; 4DP = 4-deoxypyridoxine. **B** Quantification of results (3 biological replicates). Error bars, SEM. ***P < 0.001 (unpaired *t*-test). *ey* > *GFP Ras*^*V12*^ *n* = 8; *ey* > *GFP Ras*^*V12*^4DP *n* = 7. **C** Eye imaginal discs from *ey* > *GFP Ras*^*V12*^ and *ey* > *GFP Ras*^*V12*^ 4DP third instar larvae stained for Mmp1 and Perlecan. 4DP induces an accumulation of Mmp1. Also note the diffuse staining of Perlecan, in line with the results in B. Panels are single slices obtained through Z-stack confocal acquisition. Scale bar, 50 µm. **D** Quantification of results on merged images of 10 slices. CTCF = Integrated Density – (Area of selected cell x Mean fluorescence of background readings). Error bars, SEM. *P < 0.05 (unpaired *t*-test). *ey* > *GFP Ras*^*V12*^ *n* = 4; *ey* > *GFP Ras*^*V12*^4DP *n* = 4. **E** DAPI-stained GFP-labeled cells floating in the hemolymph in *ey* > *GFP* control and *ey* > *GFP Ras*^*V12*^ larvae fed 4DP (*ey* > *GFP* control larvae express only the GFP in clones of eye-antennal discs). Scale bar, 50 µm. **F** Quantification of results. Error bars, SEM. ***P < 0.001 (chi square test). *ey* > *GFP n* = 50; *ey* > *GFP* 4DP *n* = 51; *ey* > *GFP Ras*^*V12*^ *n* = 46; *ey* > *GFP Ras*^*V12*^ 4DP *n* = 52.

Basement membrane (BM) degradation represents a crucial early step in the onset of cell spreading and metastasis [50]. Thus, to further confirm the association between PLP deficiency and secondary tumors, we assayed the BM integrity in eye discs from *Ras*^*V12*^ 4DP-fed larvae. The immunostaining with an anti-Perlecan antibody which represents a major BM component [51] yielded localized loss of Perlecan (Fig. 2A, B). Moreover, *Ras*^*V12*^ 4DP eye discs displayed an accumulation of Mmp1, a matrix metalloproteinase involved in BM breakdown [50] (Fig. 2C, D and S3), thus suggesting that vitamin B6 deficiency enhances the BM breakdown to facilitate the spreading of transformed cells.

It has been shown in *Drosophila* that cancer cells can move from the primary tumor site and travel through the hemolymph (a fluid analogous to blood) to reach secondary sites [52].

Consistently, we found that 54% of *Ras*^*V12*^ 4DP-fed larvae displayed GFP cells into the hemolymph (Fig. 2E, F). In contrast, GFP cells were found only in the hemolymph of 8% of *Ras*^*V12*^ larvae reared on a control diet and never found in control larvae (expressing only the GFP) reared on 4DP or on a control diet. By considering that not all the cells floating in the hemolymph are able to settle in distant sites [52] these results are in line with those of VNC invasions (Fig. 1C, D) and suggest that 4DP feeding can favor the migration of GFP cells through the hemolymph, allowing those who find suitable conditions to colonize distant sites.

### PLP deficiency exacerbates the cancer phenotype of *Ras*^*V12*^*Dlg*^*RNAi*^ tumors

**Fig. 3:**
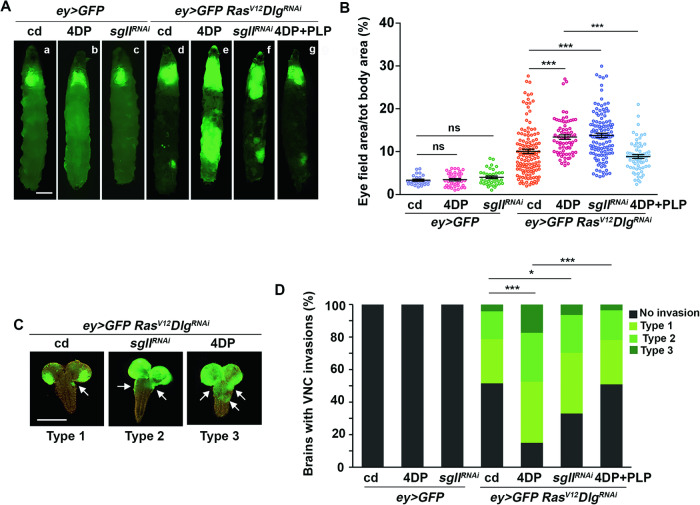
PLP deficiency increases proliferation and malignancy of *Ras*^*V12*^*Dlg*^*RNAi*^ tumors. **A** *ey* > *GFP Ras*^*V12*^*Dlg*^*RNAi*^ and control larvae reared on cd (a,d), 4DP (b,e) or 4DP + PLP(g). Larvae in c and f panels also carry the hairpin RNAi construct of the *sgll*^*PNPO*^ gene. Scale bar, 0.5 mm. cd=control diet; 4DP = 4-deoxypyridoxine; PLP=pyridoxal 5’-phosphate. (*Ras*^*V12*^*Dlg*^*RNAi*^ larvae express the oncogenic *Ras*^*V12*^ in all eye disc cells, the hairpin RNAi construct of the *Dlg* gene and the GFP construct; *ey* > *GFP* control larvae express in all eye disc cells only the GFP construct). Ectopic expression of *Ras*^*V12*^ combined to *Dlg* silencing induces tumor growth (d) exacerbated by 4DP feeding (e). RNAi mediated silencing of *sgll*^*PNPO*^gene also enhances tumor phenotype (f). **B** Percentage of GFP-positive eye field area relative to total body area. Error bars, SEM. ***P < 0.001, ns=not significant (*ey* > *GFP* cd vs *ey* > *GFP* 4DP, P = 0.795; *ey* > *GFP* cd vs *ey* > *GFP Sgll*^*RNAi*^, P = 0.1353) (unpaired *t*-test). *ey* > *GFP* cd *n* = 23; *ey* > *GFP* 4DP *n* = 37; *ey* > *GFP sgll*^*RNAi*^ *n* = 37; *ey* > *GFP Ras*^*V12*^ *Dlg*^*RNAi*^ cd *n* = 126; *ey* > *GFP Ras*^*V12*^ *Dlg*^*RNAi*^ 4DP *n* = 69; *ey* > *GFP Ras*^*V12*^ *Dlg*^*RNAi*^ *sgll*^*RNAi*^ *n* = 120; *ey* > *GFP Ras*^*V12*^ *Dlg*^*RNAi*^ 4DP + PLP *n* = 66. **C** Brains from *ey* > *GFP Ras*^*V12*^*Dlg*^*RNAi*^ larvae reared on cd or carrying the RNAi construct of the *sgll*^*PNPO*^ gene, were assigned to one of three categories based on the degree of VNC invasion. VNC= ventral nerve cord. Scale bar, 100 µm. **D** Quantification of results. The green-labeled portion of each column represents the percentage of brains with VNC invasions. The black portion the percentage of brains without invasions. The three different types of green represent arbitrary levels of invasion exemplified in **C**. Type1= mild phenotype; Type2=moderate phenotype; Type3=severe phenotype. Statistics was assessed by chi square test and refers to the percentage of invasion phenotype *P < 0.05,*** P < 0.001. The percentage of Type 3 brains from *ey* > *GFP Ras*^*V12*^*Dlg*^*RNAi*^ 4DP larvae resulted statistically significant compared to Type 3 brains from *ey* > *GFP Ras*^*V12*^*Dlg*^*RNAi*^ larvae (P = 0.0109). The percentage of Type 3 brains from *ey* > *GFP Ras*^*V12*^*Dlg*^*RNAi*^ 4DP + PLP larvae resulted statistically significant compared to Type 3 brains from *ey* > *GFP Ras*^*V12*^*Dlg*^*RNAi*^ 4DP larvae (P = 0.0143) and not significant (p = 0.85) vs *Ras*^*V12*^*Dlg*^*RNAi*^ larvae. *ey* > *GFP* cd *n* = 45; *ey* > *GFP* 4DP *n* = 48; *ey* > *GFP sgll*^*RNAi*^ *n* = 52; *ey* > *GFP Ras*^*V12*^*Dlg*^*RNAi*^ *n* = 70; *ey* > *GFP Ras*^*V12*^*Dlg*^*RNAi*^ 4DP *n* = 80; *ey* > *GFP Ras*^*V12*^*Dlg*^*RNAi*^ *sgll*^*RNAi*^ *n* = 108; *ey* > *GFP Ras*^*V12*^*Dlg*^*RNAi*^ 4DP + PLP *n* = *55*.

To confirm the role of vitamin B6 deficiency in cancer, we used also another fly cancer model (Ras^V12^ Dlg^RNAi^), in which the expression of *Ras*^*V12*^ in the entire eye-antennal disc, combined with the RNAi-induced silencing of *Discs large* (*Dlg*) polarity gene, produces neoplastic tumors able to metastasize [41]. 4DP feeding further enlarged the cephalic area of *Ras*^*V12*^ *Dlg*^*RNAi*^ larvae compared to control diet (13.4% vs 10%) (Fig. 3A, B) and produced VNC invasions in 85% of brains (vs 48.6%) (Fig. 3C, D). 4DP induced a more aggressive phenotype compared to the control diet, yielding a higher percentage of severe (Type 3) VNC invasions (Fig. 3C). In contrast, PLP feeding significantly reduced the phenotype (Fig. 3 A, B, D) In addition, we genetically induced PLP deficiency by silencing the *sgll*^*PNPO*^ gene involved in PLP biosynthesis [53]. Consistent with the effect produced by 4DP, we found an increased cephalic area (13.8%) and VNC invasion in 66.6% of brains (Fig. 3D).

Overall, these data indicate that PLP deficiency also impacts on this second cancer model, by specifically increasing the malignancy of *Ras*^*V12*^*Dlg*^*RNAi*^ tumors and triggering tumor progression.

### PLP deficiency causes chromosome damage in *Ras*^*V12*^ eye discs

**Fig. 4:**
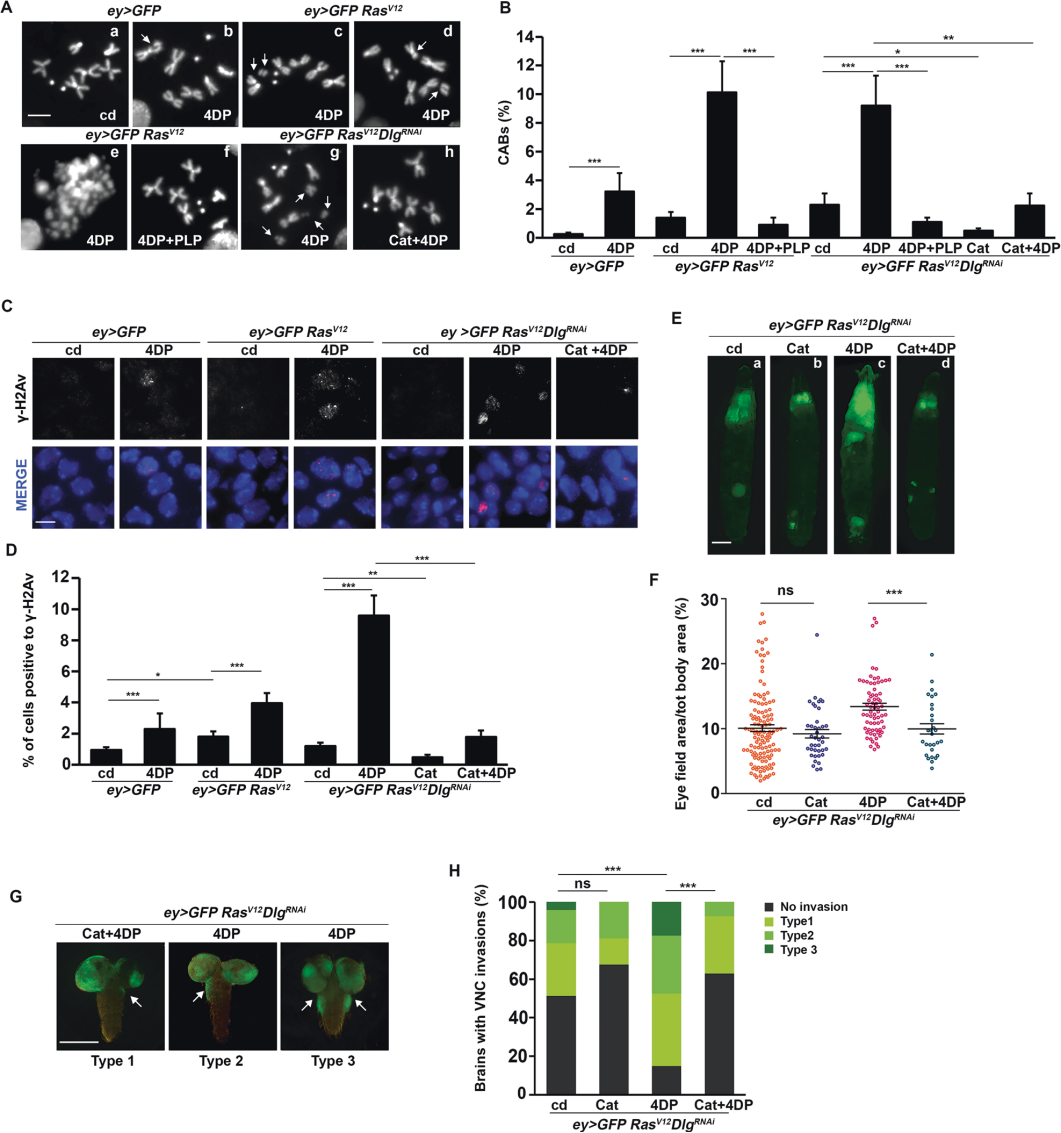
PLP deficiency causes CABs and accumulation of γ-H2Av foci in *Ras*^*V12*^ and *Ras*^*V12*^*Dlg*^*RNAi*^ eye discs. **A** Examples of chromosome aberrations (CABs) in eye discs from larvae of indicated genotypes (upper) and treatments (in the pictures). (a) Normal female metaphase; (b) chromatid deletion of a major autosome; (c) isochromatid deletion of the X chromosome (d) isochromatid deletion of a major autosome; (e) metaphase with multiply fragmented chromosomes; (f) normal female metaphase (g) metaphase with some fragmented chromosomes (h) normal female metaphase. Scale bar, 5 µm. cd=control diet; 4DP = 4-deoxypyridoxine; PLP=pyridoxal 5’-phosphate; Cat=UAS-Catalase. **B** Quantification of results. Error bars, SEM. *P < 0.05;**P < 0.01; ***P < 0.001 (chi square test). Total number of examined cells in at least three independent experiments: *ey* > *GFP* cd *n* = 771 (14 discs); *ey* > *GFP* 4DP *n* = 468 (11 discs); *ey* > *GFP Ras*^*V12*^cd *n* = 641 (11 discs); *ey* > *GFP Ras*^*V12*^ 4DP *n* = 642 (7 discs); *ey* > *GFP Ras*^*V12*^ 4DP + PLP *n* = 988 (7 discs); *ey* > *GFP Ras*^*V12*^*Dlg*^*RNAi*^cd *n* = 339 (4 discs); *ey* > *GFP Ras*^*V12*^*Dlg*^*RNAi*^ 4DP *n* = 262 (5 discs); *ey* > *GFP Ras*^*V12*^*Dlg*^*RNAi*^ 4DP + PLP *n* = 559 (5 discs); *ey* > *GFP Ras*^*V12*^*Dlg*^*RNAi*^*Cat n* = 407 *(3 discs); ey* > *GFP Ras*^*V12*^*Dlg*^*RNAi*^*Cat 4DP n* = 223 (6 discs). **C** Examples of γ-H2Av foci in eye disc nuclei from larvae of indicated genotypes and treatments. Scale bar, 5 µm. **D** Quantification of γ-H2Av positive nuclei. Error bars, SEM. *P < 0.05; **P < 0.01; ***P < 0.001 (unpaired *t*-test). *ey* > *GFP* cd *n* = 2027 (3 discs); *ey* > *GFP* 4DP *n* = 2877 (3 discs); *ey* > *GFP Ras*^*V12*^ cd *n* = 3008 (3 discs); *ey* > *GFP Ras*^*V12*^ 4DP *n* = 1464 (3 discs); *ey* > *GFP Ras*^*V12*^*Dlg*^*RNAi*^ cd *n* = 3767 (4 discs); *ey* > *GFP Ras*^*V12*^*Dlg*^*RNAi*^ 4DP *n* = 3325 (3 discs); *ey* > *GFP Ras*^*V12*^*Dlg*^*RNAi*^*Cat n* = 2297 (3 discs); *ey* > *GFP Ras*^*V12*^*Dlg*^*RNAi*^*Cat 4DP n* = 2865 (3 discs). **E** *ey* > *GFP Ras*^*V12*^*Dlg*^*RNAi*^ larvae that express or do not express *UAS-Cat* reared on cd or 4DP. Scale bar, 0.5 mm. **F** Quantification of GFP-positive eye field area relative to total body area. Error bars, SEM. ***P < 0.001, (unpaired *t*-test). ns=not significant (P = 0.392). *ey* > *GFP Ras*^*V12*^*Dlg*^*RNAi*^cd *n* = 126; *ey* > *GFP Ras*^*V12*^*Dlg*^*RNAi*^*Cat n* = 40*; ey* > *GFP Ras*^*V12*^*Dlg*^*RNAi*^*4DP n* = 69; *ey* > *GFP Ras*^*V12*^*Dlg*^*RNAi*^*Cat 4DP n* = 29. **G** Examples of brains assigned to one of three categories based on the degree of VNC invasion. Scale bar,100 µm. **H** Quantification of results. The green-labeled portion of each column represents the percentage of brains with VNC invasions. The black portion the percentage of brains without invasions. The three different types of green represent arbitrary levels of invasion. Type1=mild phenotype; type2=moderate phenotype; type 3=severe phenotype. Statistics was assessed by chi square test and refers to the percentage of invasion phenotype. *** P < 0.001. ns=not significant (P = 0.108). *ey* > *GFP Ras*^*V12*^*Dlg*^*RNAi*^ *n* = 70; *ey* > *GFP Ras*^*V12*^*Dlg*^*RNAi*^*Cat n* = 37; *ey* > *GFP Ras*^*V12*^*Dlg*^*RNAi*^ 4DP *n* = 80; *ey* > *GFP Ras*^*V12*^*Dlg*^*RNAi*^ *Cat* 4DP *n* = 27.

PLP depletion causes chromosome aberrations (CABs) in *Drosophila*, yeast, and human cells [35, 34, 54]. As CABs are well-known hallmarks of tumor initiation and progression [55], genome instability could play a major role in cancer induced by PLP deficiency. To test the validity of this hypothesis, we firstly examined eye discs from *Ras*^*V12*^ 4DP-fed larvae for the presence of chromosome damage in DAPI-stained preparations, finding 10.2% of CABs vs 1.4% in untreated *Ras*^*V12*^ larvae (Fig. 4A, B). Of note, 4DP treatment combined to *Ras*^*V12*^ expression produced a higher effect (10.2%) than the sum of the individual conditions (4DP 3%; *Ras*^*V12*^ 1,4%) (Chi square test, P < 0.001), thus suggesting that chromosome damage in *Ras*^*V12*^ 4DP discs may result from a synergistic interaction between PLP deficiency and the activated Ras (Fig. 4B). CABs were also found in *Ras*^*V12*^*Dlg*^*RNAi*^ eye discs from larvae reared in 4DP and were rescued by PLP (Fig. 4A, B).

Consistent with the notion that CABs result from improperly repaired DNA double strand breaks (DSBs) [56], 4DP treatment produced an accumulation γ-H2Av, a marker of DSBs [57], in both *Ras*^*V12*^ and *Ras*^*V12*^*Dlg*^*RNAi*^ tumor cells (Fig. 4 C, D).

PLP treatment in addition to rescue tumor development (Figs. 1 and 3) also rescued CABs in eye discs from *Ras*^*V12*^ and *Ras*^*V12*^*Dlg*^*RNAi*^ 4DP-fed larvae (Fig. 4 A, B). More interestingly, overexpression of Catalase involved in endogenous ROS scavenging [58] rescued 4DP-induced CABs, DSBs and tumors in the *Ras*^*V12*^*Dlg*^*RNAi*^ model (Fig. 4 and S4), thus providing robust indication that CABs may play a causative role in tumors induced by PLP depletion.

### PLP deficiency causes ROS accumulation and reduced activity of SHMT in *Ras*^*V12*^ eye discs

**Fig. 5:**
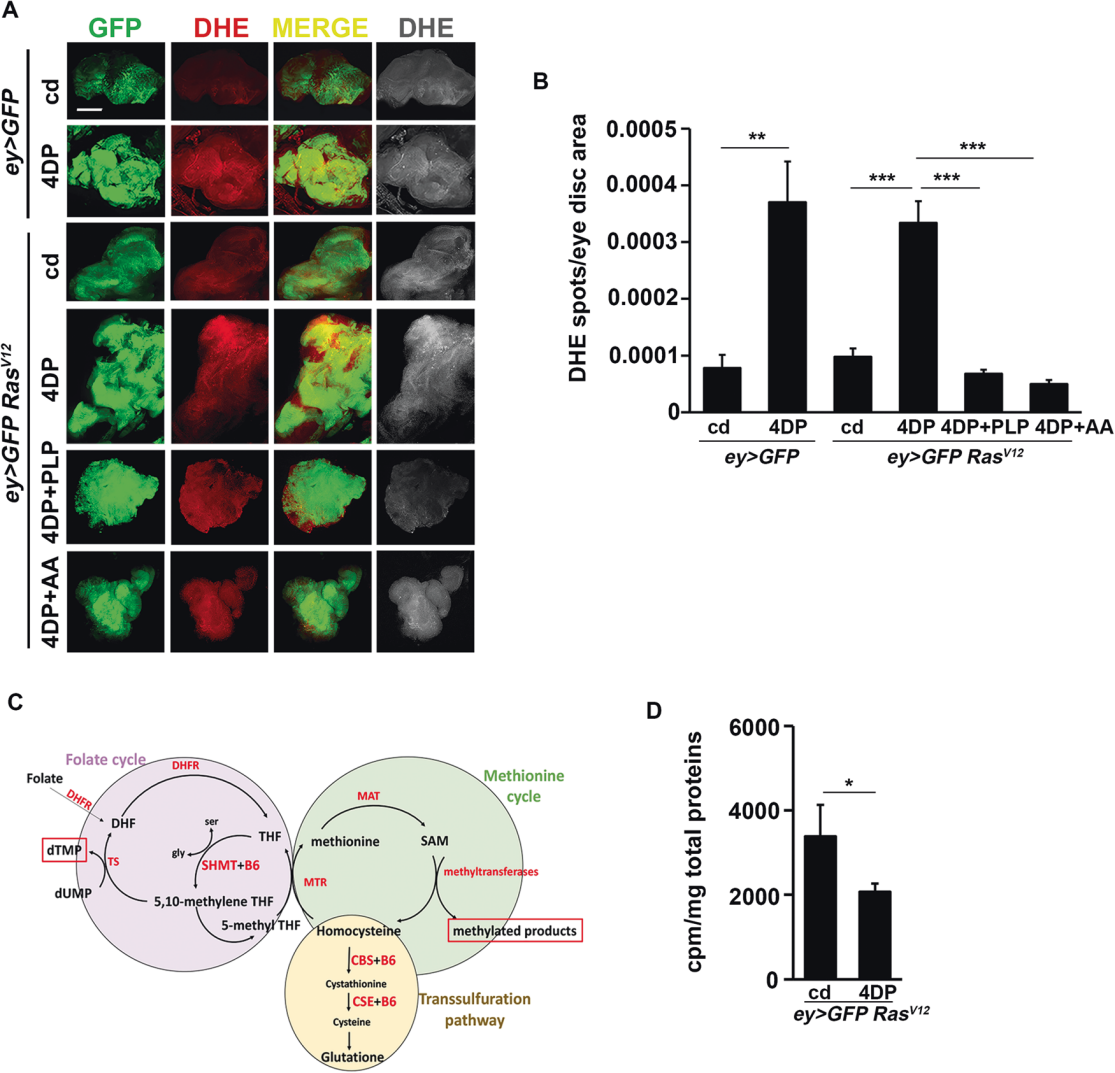
PLP deficiency causes ROS formation and reduced activity of SHMT in *Ras*^*V12*^ eye discs. **A** ROS accumulation in eye discs from *ey* > *GFP* control larvae reared on cd or 4DP and from *ey* > *GFP Ras*^*V12*^ larvae reared on cd, 4DP, 4DP + PLP or 4DP + AA. ROS were detected by the dihydroethidium (DHE) indicator dye in three independent experiments. Images were obtained through Z-stack confocal acquisition. Scale bar, 20 µm. cd=control diet; 4DP = 4-deoxypyridoxine; AA=ascorbic acid; PLP=pyridoxal-phosphate. **B** Quantitative analysis of DHE positive puncta/eye disc area performed with the ImageJ software on acquired images. Error bars, SEM. **P < 0.01; ***P < 0.001 (unpaired *t*-test). *ey* > *GFP* cd *n* = 11; *ey* > *GFP* 4DP *n* = 13; *ey* > *GFP Ras*^*V12*^cd *n* = 13*; ey* > *GFP Ras*^*V12*^ 4DP *n* = 22; *ey* > *GFP Ras*^*V12*^ 4DP + PLP *n* = 18; *ey* > *GFP Ras*^*V12*^ 4DP + AA *n* = 23. **C** Simplified scheme of one carbon metabolism. The PLP-dependent serine hydroxymethyltransfeRase (SHMT) converts tetrahydrofolate (THF) to 5,10-methylenetetrahydrofolate. 5,10-methylenetetrahydrofolate is in part utilized to produce thymidylate (dTMP) and in part to produce methionine and hence S-Adenosyl methionine SAM, in turn used by methyltransfeRases to methylate their substrates. CBS= cystathionine ß-synthase; CSE=cystathionine γ‐lyase; DHFR=dihydrofolate reductase; MAT=methionine adenosyltransfeRases; MS=methionine synthase; MTHFR=methylenetetrahydrofolate reductase; SHMT=serine hydroxymethyltransfeRase; TS= thymidylate synthase. **D** SHMT catalytic activity in eye discs from *ey* > *GFP Ras*^*V12*^ 4DP larvae. Error bars, SEM. *P < 0.05 (unpaired *t*-test). *ey* > *GFP Ras*^*V12*^ cd *n* = 100; *ey* > *GFP Ras*^*V12*^4DP *n* = 100.

We previously demonstrated in *Drosophila* that CABs resulting from PLP deficiency derive in part from ROS accumulation and in part from decreased dTMP availability, in turn caused by reduced SHMT catalytic activity [35, 36]. This is consistent with the role of vitamin B6 as antioxidant molecule [59–62] and with its role as a cofactor of SHMT, whose reaction yields 1 C units that ultimately produce thymidylate (dTMP) required for DNA synthesis and repair [3]. To investigate if the origin of CABs was the same also in PLP deficient *Ras*^*V12*^ cells, we examined eye discs from *Ras*^*V12*^ 4DP-fed larvae by evaluating ROS accumulation and measuring the catalytic activity of SHMT. As shown in Fig. 5 A, B, dihydroethidium (DHE) staining revealed that eye discs from *Ras*^*V12*^ 4DP-fed larvae accumulated, in GFP-labeled clones, more ROS compared to *Ras*^*V12*^ discs from larvae reared on a control diet. In contrast, feeding *Ras*^*V12*^ 4DP-treated larvae with the antioxidant ascorbic acid (AA) or PLP counteracted the formation of ROS (Fig. 5A, B). In addition, eye discs from 4DP-fed *Ras*^*V12*^ larvae displayed a reduced catalytic activity of SHMT, compared to discs from *Ras*^*V12*^ larvae fed a standard diet (Fig. 5D and S5). Given the role of SHMT in folate cycle this result suggests that discs from *Ras*^*V12*^ 4DP-fed larvae undergo a reduced biosynthesis of dTMP (Fig. 5C).

### Role of oxidative stress and reduced activity of SHMT in tumors induced by PLP deficiency

**Fig. 6:**
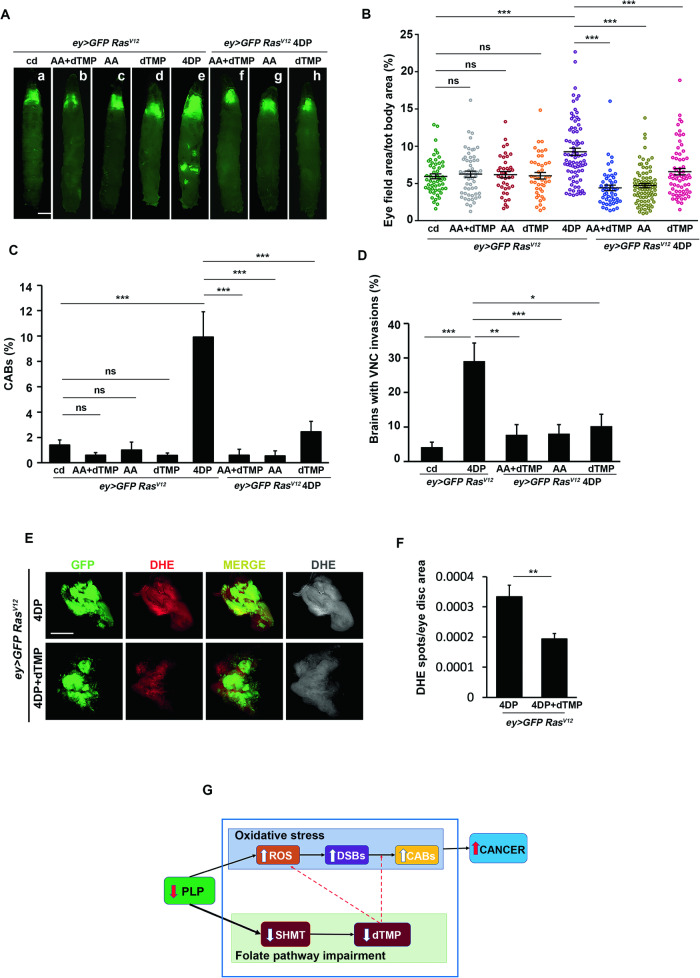
The combined effect of ROS accumulation and dTMP depletion promotes both genome instability and cancer development in *Ras*^*V12*^ 4DP eye discs. **A** *ey* > *GFP Ras*^*V12*^ and *ey* > *GFP Ras*^*V12*^4DP-fed larvae reared on cd medium (a,e) or on media containing AA+dTMP (b,f), AA (c,g) or dTMP (d,h). Scale bar, 0.5 mm. **B** Percentage of GFP-positive eye field area relative to total body area. Error bars, SEM. ***P < 0.001, ns=not significant. *ey* > *GFP Ras*^*V12*^ AA+dTMP vs *ey* > *GFP Ras*^*V12*^ cd P = 0.56; *ey* > *GFP Ras*^*V12*^ AA vs *ey* > *GFP Ras*^*V12*^cd P = 0.94; *ey* > *GFP Ras*^*V12*^ dTMP vs *ey* > *GFP Ras*^*V12*^cd P = 0.50 (unpaired *t*-test). Data are representative of at least three different experiments*. ey* > *GFP Ras*^*V12*^ cd *n* = 54*; ey* > *GFP Ras*^*V12*^ AA+dTMP *n* = 55*; ey* > *GFP Ras*^*V12*^ AA *n* = 38*; ey* > *GFP Ras*^*V12*^ dTMP *n* = 43; *ey* > *GFP Ras*^*V12*^ 4DP *n* = 78; *ey* > *GFP Ras*^*V12*^ 4DP AA+dTMP *n* = 48; *ey* > *GFP Ras*^*V12*^ 4DP AA *n* = 81; *ey* > *GFP Ras*^*V12*^ 4DP dTMP *n* = 66. cd=control diet; 4DP = 4-deoxypyridoxine; AA=ascorbic acid; dTMP=deoxythymidine monophosphate. **C** CAB frequency in eye discs from *ey* > *GFP Ras*^*V12*^ larvae reared on cd or 4DP medium supplemented with AA+dTMP, AA or dTMP. ***P < 0.001, ns=not significant. *ey* > *GFP Ras*^*V12*^ AA+dTMP vs *ey* > *GFP Ras*^*V12*^ P = 0.11; *ey* > *GFP Ras*^*V12*^ AA vs *ey* > *GFP Ras*^*V12*^ P = 0.59; *ey* > *GFP Ras*^*V12*^ dTMP vs *ey* > *GFP Ras*^*V12*^P = 0.062 (chi square test). Total number of examined cells in at least three different experiments: *ey* > *GFP Ras*^*V12*^ cd *n* = 641 (11 discs)*; ey* > *GFP Ras*^*V12*^ AA+dTMP *n* = 840 (5 discs)*; ey* > *GFP Ras*^*V12*^ AA *n* = 908 (5 discs)*; ey* > *GFP Ras*^*V12*^ dTMP *n* = 1368 (7 discs); *ey* > *GFP Ras*^*V12*^ 4DP *n* = 383 (4 discs); *ey* > *GFP Ras*^*V12*^ 4DP AA+dTMP *n* = 1345 (11 discs); *ey* > *GFP Ras*^*V12*^ 4DP + AA *n* = 371 (5 discs); *ey* > *GFP Ras*^*V12*^ 4DP dTMP *n* = 856 (6 discs). **D** Quantification of VNC invasion in brains from *ey* > *GFP Ras*^*V12*^ 4DP-fed larvae reared on AA+dTMP, AA, or dTMP media. Error bars, SEM in at least three independent experiments *P < 0.05, **P < 0.01, ***P < 0.001 (chi square test). *ey* > *GFP Ras*^*V12*^ cd *n* = 48; *ey* > *GFP Ras*^*V12*^ 4DP *n* = 55; *ey* > *GFP Ras*^*V12*^ 4DP AA+dTMP *n* = 52 *ey* > *GFP Ras*^*V12*^ 4DP AA *n* = 50; *ey* > *GFP Ras*^*V12*^ 4DP dTMP *n* = 49. **E** ROS accumulation in eye discs from *ey* > *GFP Ras*^*V12*^ 4DP larvae and *ey* > *GFP Ras*^*V12*^ 4DP supplemented with dTMP. ROS were detected by the dihydroethidium (DHE) indicator dye in three independent experiments. Images were obtained through Z-stack confocal acquisition. Scale bar, 20 µm. **F** Quantitative analysis of DHE positive puncta/eye disc area performed with the ImageJ software on acquired images. dTMP treatment reduced the number of DHE positive spots. Error bars, SEM, **P < 0.01 (unpaired *t*-test). *ey* > *GFP Ras*^*V12*^ 4DP *n* = 22 *ey* > *GFP Ras*^*V12*^ 4DP dTMP *n* = 22. **G** Scheme illustrating the putative mechanism at the basis of cancer induced by PLP deficiency. Low PLP levels increase oxidative stress and produces DNA double strand breaks (DSBs) that are converted into to CABs, which increase the cancer risk. PLP deficiency also reduces the SHMT catalytic activity which, in turn, reduces the biosynthesis of dTMP. dTMP depletion may impact on ROS-induced DSBs and increase ROS production, thus amplifying genome instability.

To better correlate the oxidative stress and the reduced activity of SHMT with tumors induced by PLP deficiency and to shed light on their relative contributes, we fed flies with AA and dTMP alone or in combination, finding a complete rescue of both CABs and primary tumors, which further confirmed the role of CABs in 4DP-induced cancers (Fig. 6A-C and S6). CAB frequency decreased from 9.92% to 0.59% (Fig. 6C), the percentage of GFP-labeled area decreased from 9.25% to 4.42% (Fig. 6A, B), and the frequency of VNC invasions decreased from 29% to 7.7% (Fig. 6D).

Feeding *Ras*^*V12*^ 4DP larvae with AA yielded a complete rescue of both chromosome damage (CABs=0.54%) (Fig. 6C and S6), primary and secondary tumors (cephalic area=4.71%; VNC invasions= 8.0%) (Fig. 6A, B, D). The same effect was obtained with alpha-lipoic acid, another strong antioxidant (Fig. S7). Supplementation with dTMP produced a less pronounced rescue of CABs with respect to AA (2.45%) (Fig. 6C and S6), and a reduction of the area occupied by primary (6.6%) (Fig. 6A, B) and secondary tumors (VNC invasions =10.2%) (Fig. 6D).

Taken together these results suggested that both SHMT depletion and ROS accumulation may have a role in CAB production induced by PLP deficiency in *Ras*^*V12*^, and also that they may synergize to increase the genome instability, thus predisposing cells to malignant transformation. Since dTMP is involved in DNA metabolism, its depletion may compromise the repair of lesions caused by ROS. Furthermore, in agreement with previous studies indicating that the knockout of SHMT2 increases ROS production in human cancer cells [63], dTMP supplementation reduced DHE staining of about 50% (Fig. 6E, F) in eye discs from *Ras*^*V12*^ 4DP-fed larvae, thus suggesting that under PLP deficiency dTMP depletion, may also contribute to increase the oxidative stress, besides compromising repair (Fig. 6G).

In conclusion, altogether, these results provide robust evidence that vitamin B6 deficiency can impact on *Ras*^*V12*^ cancers and that this effect may be mediated by increased genome instability, in turn produced by the concerted action of ROS accumulation and decreased dTMP biosynthesis.

## Discussion

Our study, carried out in *Drosophila*, provides the first compelling evidence that vitamin B6 deficiency affects cancer by promoting genome instability. In addition, it identifies two main mechanisms by which genome damage is produced in PLP deficient cells: increased ROS formation and reduced SHMT activity.

Several studies inversely correlated micronutrient plasma levels and human cancer risk. However, although many micronutrients are potential cancer promoting candidates because of their functional and biochemical properties, the establishing of a precise cause-effect relationship between diet and cancer in both observational epidemiological studies and intervention trials has proved challenging [64]. Using animal models may enable not only to establish these relationships more clearly, but also to identify underlying molecular mechanisms.

Among the animal cancer models, the common fruit fly is emerging as a precious resource for cancer biology and metabolic studies [65], since it offers a valuable toolkit with various advantages including high genetic conservation of major metabolic and cancer pathways and similar drug response to mammals.

Here we have shown that PLP deficiency, induced by 4DP feeding, is able to transform benign *Ras*^*V12*^ cancers in more aggressive forms capable of generating secondary tumors as demonstrated by (i) migration of GFP cells into the brain VNC, (ii) membrane basement degradation, (iii) accumulation of Mmp1, and (iv) presence of GFP cells into the hemolymph of *Ras*^*V12*^ 4DP-fed larvae. The finding that even ginkgotoxin (GT), another antagonist of PLP [66], is able to transform *Ras*^*V12*^ tumors further corroborates our results.

In addition, we have shown that PLP deficiency can also impact on the *Ras*^*V12*^ *Dlg*^*RNAi*^ cancers, worsening their aggressiveness. Interestingly, the specific depletion of Sgll^/PNPO^ in *Ras*^*V12*^*Dlg*^*RNAi*^ eye discs displayed the same effect as 4DP feeding, allowing us to exclude a major role of systemic effects of 4DP on cancer phenotypes.

Although several studies were focused on the involvement of vitamin B6 in cancer [10, 22], the underlying mechanisms are still elusive. We and other authors have previously demonstrated that PLP deficiency yields CABs and micronuclei in *Drosophila* and human cells, respectively, thus suggesting that genome instability may mediate the role of PLP in cancer [35, 54]. CABs can, indeed, provide growth benefits to precancerous cells or promote cancer progression by impacting on the formation of hybrid genes or the deletion of tumor suppressor genes [67, 68]. Here we have shown that eye discs from *Ras*^*V12*^ 4DP-fed larvae displayed CABs, accumulated ROS and exhibited a reduced SHMT catalytic activity, in line with previous studies indicating that CABs originate from oxidative stress and reduced dTMP biosynthesis in PLP deficient cells [35, 36]. Additionally, we provided robust evidence that CABs represent a major cause of tumors in PLP deficient larvae. By acting on the mechanisms that generate CABs in PLP depleted cells (such as ROS accumulation and dTMP depletion), we fed larvae with a diet made of AA+dTMP, finding a rescue of both CABs and tumors. Furthermore, overexpressing the *Catalase* gene in *Ras*^*V12*^*Dlg*^*RNAi*^ tumor cells we also found that both 4DP-induced CABs and tumors were strongly reduced.

To investigate the relative roles of ROS accumulation and SHMT depletion in 4DP-induced tumors, we have supplied AA and dTMP separately. The complete rescue obtained with AA diet suggests that dTMP works downstream to ROS or, alternatively, that dTMP biosynthesis plays only a modest role. However, the finding that dTMP diet can rescue CABs and tumors would favor the first hypothesis. Thus, based on the role of dTMP in DNA synthesis and repair and on our finding that dTMP diet decreases ROS accumulation, we propose the following model that links, PLP deficiency, CABs, and cancer together (Fig. 5G).

Due to the ability of PLP to scavenge ROS both directly and indirectly [8], in PLP-deficient cells, weakened cellular antioxidant defenses, would increase the production of ROS, which attack DNA leading to the formation of DSBs, thus predisposing cells to cancer. Meanwhile, a reduced availability of PLP cofactor would reduce the biosynthesis of dTMP by affecting the catalytic activity of SHMT, thus amplifying the oxidative stress by both altering the repair of DNA ROS-induced lesions and increasing the production of ROS. Regarding the effect of dTMP depletion on the ROS increase, we do not have a molecular explanation, however our results are consistent with studies showing that inhibitors of thymidylate synthase, the enzyme directly involved in dTMP biosynthesis (Fig. 5C), can increase intracellular ROS by activating the enzyme NADPH oxidase and promoting apoptosis [69]. Similarly, the mitochondrial SHMT2 knockdown in bladder cancer cells leads to ROS accumulation and apoptosis [63]. We can, therefore, hypothesize that, in our model, 4DP treatment causes a less severe reduction of dTMP synthesis compared to SHMT knockdown or to a TS inhibition, thus allowing some cells with DNA damage to escape apoptosis and go towards cancer transformation.

Although we did not examine secondary tumors for chromosome damage, the finding that AA and dTMP diets alone or in combination rescue the phenotype may anyhow suggest that genome instability triggered by the concerted action of dTMP depletion and ROS increase may also contribute to the spread of tumor cells.

In this work, we considered only CABs and DSBs as genome damage endpoint, however we expect that a part of genome instability depends also on mutations due to DNA base oxidation (such as 8-oxo-2’-deoxyguanosine, 8 oxo-dG) and erroneous incorporation of dUTP into DNA. In line with this hypothesis, vitamin B6 in mice significantly suppressed colon cancer induced by azoxymethane (AOM) by decreasing levels of oxidative stress markers including 8 oxo-dG [31]. Moreover, cytosolic SHMT1 knockdown increased dUTP incorporation in lung cancer cells [70].

Finally, we can also hypothesize that the decreased catalytic activity of SHMT due to PLP deficiency may also alter the chromatin methylation patterns, thus contributing to *Ras*^*V12*^ transformation [71]; this hypothesis will be tested in future studies.

In conclusion, we were able to demonstrate, for the first time, that PLP deficiency triggers cancer development in *Drosophila* and more importantly, we identified genome instability as an important player. Although vitamin B6 is present in most foods and hence an overt deficiency of this vitamin is rare, reduced PLP levels are associated with several common pathologies including diabetes and malabsorption syndromes [8]. Thus, applied to humans, our results may suggest the importance of evaluating the genome integrity as a biomarker of cancer risk factor in all the contexts of reduced availability of vitamin B6.

## Materials and methods

### Fly stocks

*y,w eyFlp; Act* > *y* + *> Gal4 UAS GFP; FRT82B, Tub Gal80* was kindly provided by T. Xu lab (Yale School of Medicine).

*UAS Ras*^*V12*^*/ UAS Ras*^*V12*^*; FRT82B/ FRT82B* was kindly provided by Hirabayashi lab (London, Institute of Medical Science).

*eyflp; UAS-Ras*^*V12*^*, UAS-Dlg*^*RNAi*^*/CyO, Gal80; act* > *CD2>Gal4, UAS-GFP* and *eyflp; Sp/CyO,Gal80; act4* > *CD2>Gal4, UAS-GFP* stocks were obtained by K. Basler (Institute of Molecular Life Sciences, University of Zurich, Switzerland). The *sgll* RNAi line (# 105941) was obtained by VDRC stock center (Vienna). The stock*w*[1]*; P{w[+mC]=UAS-Cat.A}2* (Bl #24621) was obtained by Bloomington Indiana Stock Center.

*Oregon-R* was used as control wild type stock.

The balancers used in this work and the genetic markers are described in detail on FlyBase (http://flybase.bio.indiana.edu/).

### Genetic Crosses

- To generate larvae with clonal *Ras*^*V1*2^ tumors in eye-antennal discs we crossed:
  *y,w eyFlp; Act* > *y* + *> Gal4 UAS GFP; FRT82B, Tub Gal80* females to *UAS Ras*^*V12*^*/ UAS Ras*^*V12*^*; FRT82B/ FRT82B* males.
- To generate control larvae expressing in clonal way only the GFP in the eye discs we crossed *y,w eyFlp; Act* > *y* + *>Gal4 UAS GFP; FRT82B, Tub Gal80* females to *Cy/Sco; FRT82B/TM6B,Tb* males.
- To generate larvae carrying *Ras*^*V12*^ *Dlg*^*RNAi*^ tumors we crossed
  *eyflp; UAS-Ras*^*V12*^*, UAS-Dlg* ^*RNAi*^*/CyO, Gal80; act* > *CD2>Gal4, UAS-GFP* females to *Oregon-R* males.
- To test the effect of *sgll*^*PNPO*^ silencing on *Ras*^*V12*^ *Dlg*^*RNAi*^ tumors, we crossed:
  *eyflp; UAS-Ras*^*V12*^*, UAS-Dlg* ^*RNAi*^*/CyO, Gal80; act* > *CD2>Gal4, UAS-GFP* females to males from the *sgll* RNAi line (VDRC # 105941).
- Control larvae expressing GFP in the entire eye-antennal disc were obtained by crossing:
  *eyflp; Sp/CyO,Gal80; act4* > *CD2>Gal4, UAS-GFP* females to *Oregon-R* males.
- Control larvae to test the effect of Sgll depletion on GFP expression were obtained by crossing *eyflp; Sp/CyO,Gal80; act4* > *CD2>Gal4, UAS-GFP* females to *sgll* RNAi males.
- To generate larvae expressing *Ras*^*V12*^ in the entire eye discs we crossed *eyflp; Sp/CyO,Gal80; act4* > *CD2>Gal4, UAS-GFP* females to *UAS Ras*^*V12V*^*/ UAS Ras*^*V12*^ males.
- To generate *Ras*^*V12*^*Dlg*^*RNAi*^ larvae overexpressing Catalase we crossed:
  *eyflp; UAS-Ras*^*V12*^*, UAS-Dlg* ^*RNAi*^*/CyO, Gal8 ; act* > *CD2>Gal4, UAS-GFP* females to *w*[1]*; P{w[+mC]=UAS-Cat.A}2* males.
- To obtain control larvae expressing Catalase in a non-tumor background we crossed *eyflp; Sp/CyO,Gal80; act4* > *CD2>Gal4, UAS-GFP* females to *w*[1]*; P{w[+mC]=UAS-Cat.A}2* males.

### Treatments

All stocks were maintained and crossed at 25 °C on a standard medium containing in 100 mL: 0.68 g agar, 6.52 g yeast, 3 g flour, 600 µL propionic acid, and 5.13 g sucrose.

PLP antagonists such as 4-deoxypyridoxine (4DP, Sigma Cat. No. D0501) and ginkgotoxin (GT, Sigma Cat. No. 89960), as well as pyridoxal 5’phosphate (PLP, Sigma Cat. No. P9255), were dissolved in the standard medium at 2 mM, 0.2 mM and 0.5 mM final concentrations, respectively. 4DP and PLP concentrations were chosen according to [35]; GT concentration according to [72].

Deoxythymidine monophosphate (dTMP, Merck Cat. No. T7004‐100MG) was dissolved in the standard medium at 200 μM concentration. This concentration was established by adapting the concentration used in in vitro experiments to the oral administration [36]. Ascorbic acid (Sigma Cat. No. A 5960) was dissolved to standard medium at a final concentration of 40 mM according to [73].

Alpha-lipoic acid (ALA, Sigma Cat. No. T1395) was added to medium at 2 mM concentration according to [35].

### Analysis of larvae

Larvae were immobilized by keeping them for at least 4 hours in PBS at 4 °C, and then examined under the fluorescence microscope (Carl Zeiss -Thornwood, NY) equipped with an HBO100W mercury lamp and a cooled charged‐coupled device (CCD camera; Photometrics CoolSnap HQ). The GFP-labeled area measurements were performed on the acquired pictures using imageJ software.

Larvae from at least 5 independent experiments have been examined.

### Analysis of isolated brains

Brains from wandering third-instar larvae were dissected in PBS, and the distribution patterns of GFP clones were examined in optical lobes and ventral nerve cords (VNCs) under the fluorescence microscope (Carl Zeiss). The invasions of GFP clones from their original sites (eye-antennal discs and optical lobes) to VNCs were considered secondary tumors. Brains from 3 independent experiments have been analyzed.

### Chromosome cytology

To analyze chromosome aberrations (CABs), eye discs from third instar larvae were dissected in saline (NaCl 0.7%). Metaphases were exposed to colchicine (final concentration 10^-5 ^M) for 1 hr. The eye discs were then incubated in the hypotonic solution (sodium citrate 0.5%) for 7 minutes, squashed in 45% acetic acid, frozen in liquid nitrogen and mounted in Vectashield H‐1200 with 4,6 diamidino‐2‐phenylindole (DAPI; Vector Laboratories, Burlingame, CA). Observations were carried out using a Zeiss Axioplan fluorescence microscope equipped with CCD camera (Photometrics CoolSnap HQ).

To assay the effect of AA, dTMP or AA+dTMP in vitro, eye discs dissected from *Ras*^*V12*^ 4DP third instar larvae were incubated in 2 ml of saline supplemented with 10% fetal bovine serum (FBS, Gibco BRL) for 4 hours with addition of 50 µM dTMP according to [36] or 5 mM AA. One hour before fixation colchicine (final concentration, 10^−5^ M) was added to the saline/FBS to collect metaphases. Discs were then fixed with the standard procedure above described.

To calculate the percentage of CABs we arbitrarily assigned only five CABs to each cell with multifragmented chromosomes.

### Immunofluorescence

Eye imaginal discs from third instar larvae were dissected in PBS and fixed in a 4% formaldehyde solution for 30 minutes on a rotating wheel at RT. Fixed tissues were then washed twice in a PBS 0,3% Triton (PBT) solution for 10 minutes and blocked in a PBT 5% Normal Donkey Serum (NDS) solution for 45 minutes. Samples were incubated overnight at 4 °C with primary antibody solution.

The day after, eye discs were washed twice in PBT and then incubated for 3 hours in the dark with. After two 10-minutes washes, samples were incubated in a DAPI solution for 30 minutes to stain nuclei. Finally, once removed tissue debris, samples were mounted onto microscope slides in FluoroMount (Sigma, F4680) medium and then sealed with nail polish.

The primary antibodies were: rabbit anti-Perlecan (1:1000 diluted in PBT 1% NDS, a gift of L. Pierre, Institute Curie, Paris, France); mouse anti Mmp1 (a cocktail of antibodies against Mmp1 catalytic domain #3B8D12 and #5H7B11, from Developmental Studies Hybridoma Bank, 1:50 diluted in PBT 1% NDS, a gift of T. Vaccari, Università degli studi di Milano, Italy).

The secondary antibodies were: donkey anti-rabbit Alexa-555-conjugated (1:600 diluted in PBT 1% NDS, Thermo Fisher Scientific Cat. No. A31572); donkey anti-mouse Alexa Fluor 555-conjugated (1:600 diluted in PBT 1% NDS, Thermo Fisher Scientific Cat. No. A32773) and donkey anti-rabbit Alexa Fluor 647-conjugated (1:600 diluted in PBT 1% NDS, Thermo Fisher Scientific Cat. No. A32795).

Immunofluorescence analysis was performed through a confocal laser scanning microscope (LSM800, Zeiss, Jena, Germany) equipped with four laser lines: 405 nm, 488 nm, 561 nm, and 639 nm. For each imaginal disc at least ten slices were acquired through Z-stack technology. The brightness and contrast of the digital images were adjusted using Zeiss Zen software 3.0 blue edition (Zeiss, Jena, Germany) and Adobe Photoshop CS6 (Adobe, San Jose, CA, USA).

The rate of BM damage was measured by analyzing each slice of each imaginal disc, scoring 120 images for each experimental condition. Fluorescence intensity of Mmp1 on eye discs was measured using the Image J software.

Immunostaining for pH3 and γ-H2Av was performed on eye discs from third instar larvae dissected and fixed as described in [74]. After several rinses in phosphate buffered saline 0.1% Triton (PBS-T) eye disc preparations were incubated overnight at 4 °C with primary antibodies diluted in PBT. After two rinses in PBT primary antibodies were detected by incubation for 1 h with the appropriate secondary antibody. We used the following primary antibodies: rabbit anti-phospho-Histone H3 (pSer10) Sigma-Aldrich Cat. No. 06570 (1:50, a gift of M. Gatti, Sapienza University of Rome, Italy) and rabbit anti-Histone H2AvD pS137 (1:100; Rockland Cat. No. 600-401-914). Secondary antibody was Alexa Fluor 555-conjugated anti-rabbit (1:300 in PBT; Thermo Fisher Scientific Cat. No. A31572). Immunostained preparations were mounted in Vectashield H-1200. Observations were carried out using a Zeiss Axioplan fluorescence microscope equipped with CCD camera (Photometrics CoolSnap HQ). The γ-H2AV positive cells were quantified on the acquired pictures using Adobe Photoshop 2022 version 23.5.5.

### Dihydroethidium (DHE) staining

To evaluate ROS accumulation, third instar larval eye-antennal imaginal discs were dissected in Schneiders medium (Gibco, Cat. No. 21720024*)* and incubated in 30uM Dihydroethidium (DHE) (Thermo Fisher Cat. No. D23107) in PBS dye solution for 5 minutes in a dark chamber, on orbital shaker at room temperature. DHE is oxidized by superoxide radical to form 2-hydroxyethidium which intercalates with DNA and provides signal at 550 nm in cells where ROS are produced [75]. After 3 washes in Schneiders medium and 1 wash in PBS on an orbital shaker at room temperature, discs were immediately mounted in DAPI Vectashield. Images were immediately captured using the confocal microscope. Quantification was performed by using ImageJ/Fiji plugin to count spots (Spot Counter Plug-in version 0.14) and was expressed as DHE positive puncta/eye disc area.

### Analysis of GFP cells into the hemolymph

Hemolymph was extracted from 20 larvae as described in [76]. 20 µL of hemolymph was mixed on a slide with 5 µL of DAPI and examined under the fluorescence microscope at 20x magnification. GFP cells were counted on acquired pictures using Photoshop software (2022 v23.5.5).

### SHMT activity measurement

Measurement of SHMT activity was performed using a radioisotope assay based on the ability of SHMT to catalyze the exchange of the pro‐2 S proton of glycine with solvent [77]. Protein extracts obtained from about 100 discs in 20 mM K‐phosphate, pH 7.2, containing 150 mM NaCl, 0.1% NP‐40 and 5 mM 2‐mercapto ethanol, were incubated with tritiated [23H] glycine (23 nmol/L) at 30 °C for 4 h and treated as previously described [78]. The experiment was repeated four times, duplicates were used each time and the radioactivity was normalized to total protein content, determined with Bradford’s assay.

### Statistical analysis

All data are expressed as mean ± standard error of the mean (SEM) from at least three independent experiments. Statistical significance was performed using the unpaired two-tailed *t-*test or the Chi square test as indicated in each figure legend. P < 0.05 was considered significant. Statistical parameters of individual experiments (value of n, mean, SEM, P values) are reported in each figure legend.

### Supplementary information

Supplementary information

## Data Availability

All data reported in this paper will be shared upon request.
