# Supplementary material for: Vitamin B6 deficiency cooperates with oncogenic Ras to induce malignant tumors in Drosophila
Source: Cell Death Dis. 2024 Jun 3;15(6):388. doi: 10.1038/s41419-024-06787-3 (PMC11148137; doi:10.1038/s41419-024-06787-3)
Supplement: Supplementary file 1 — Supplementary information [file 41419_2024_6787_MOESM1_ESM.docx]

­­­­­­
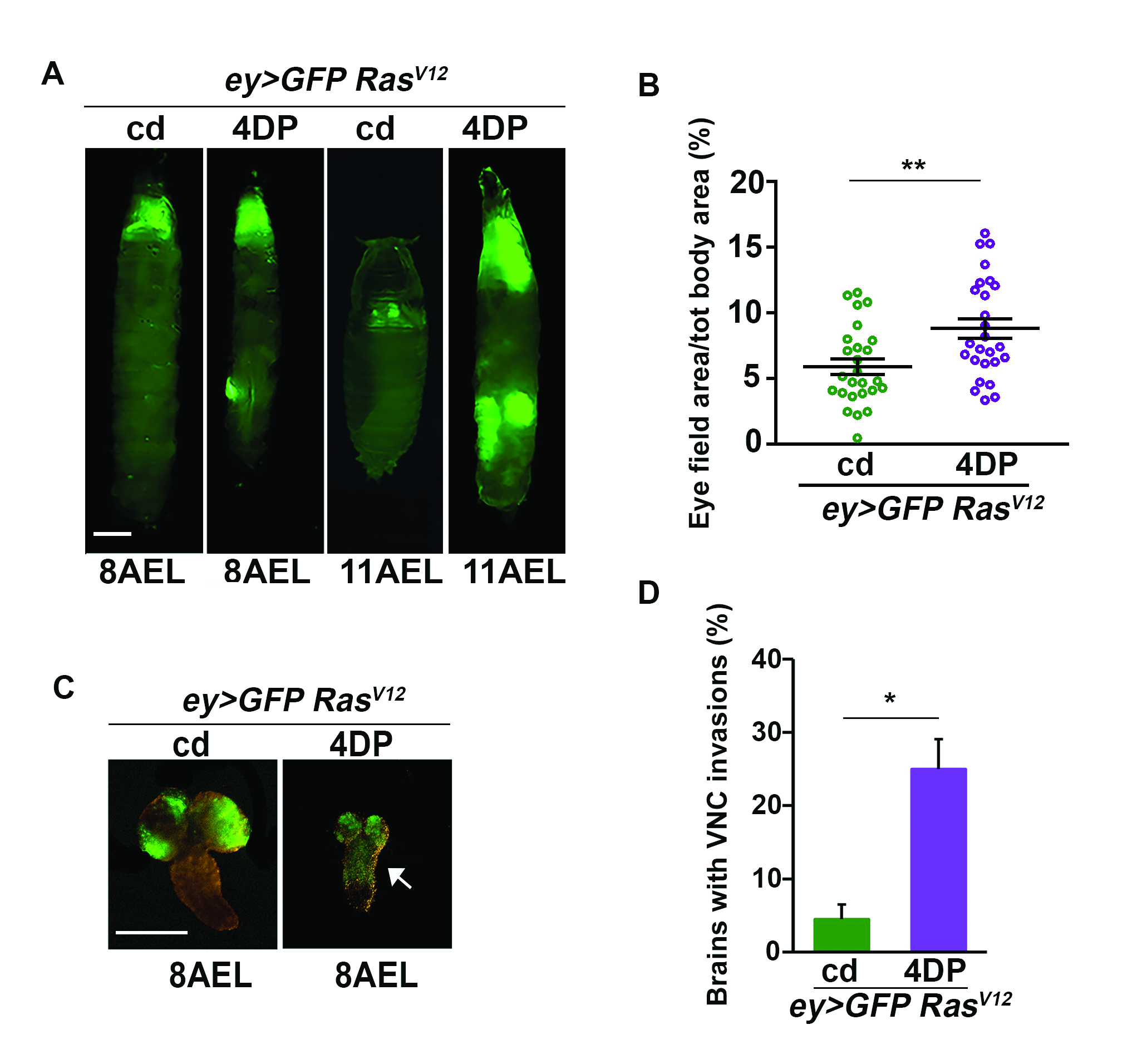
­­

**Figure S1** **A** Developmental-stage-matched third-instar larvae reared on a control diet (cd) or on 4-deoxypyridoxine (4DP) Scale bar, 0.5mm. At day 8 after egg laying (AEL) larval body was significantly smaller in 4DP-fed animals than in age-matched controls, but the percentage of cephalic area occupied by GFP-positive clones was proportionally significantly larger than controls. **B** Quantification of GFP-positive eye field area relative to total body area. Bar errors, SEM. **P<0.01 (unpaired *t*-test). *ey>GFP Ras^V12^* cd *n*=26; *ey>GFP* *Ras^V12^* 4DP *n*=26.

**C** At day 8 AEL *ey>GFP* *Ras^V12^* 4DP-fed larvae already display VNC invasions (arrow). This indicates that tumor phenotype shown by *ey>GFP* *Ras^V12^* 4DP larvae at day 11 AEL is not due to their older larval age. Scale bar, 100µm. **D** Quantification of VNC invasions. Bar errors, SEM. * P<0.05 (chi square test). *ey>GFP Ras^V12^* cd *n*=23; *ey>GFP Ras^V12^* 4DP *n*=28.


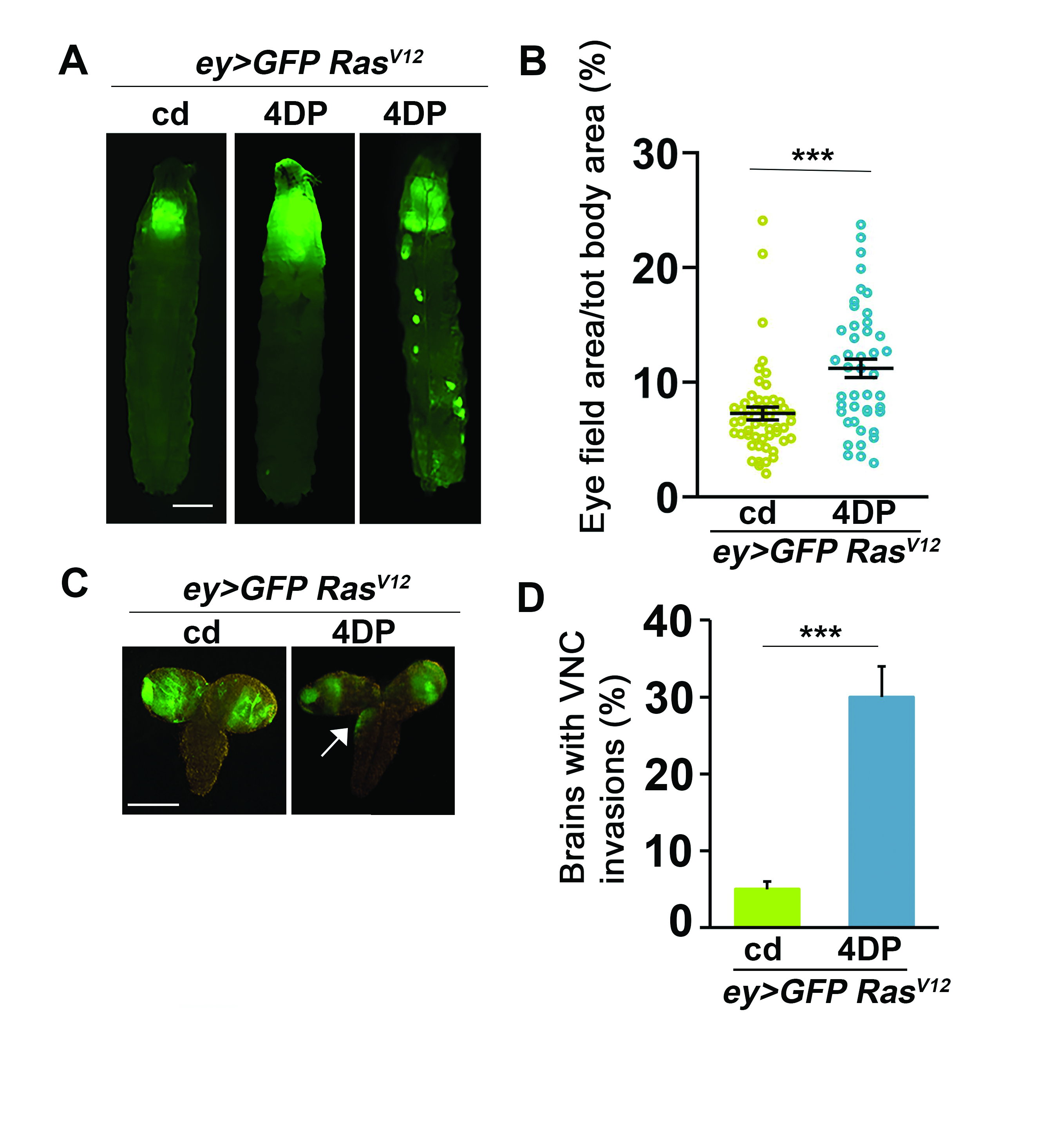


**Figure S2** PLP deficiency induced by 4DP causes tumor phenotypes also in a model expressing *Ras^V12^* in the entire eye disc. **A** Examples of *ey>GFP* *Ras^V12^* larvae reared on a control diet (cd) or on a 4DP diet (4DP). Scale bar, 0.5mm **B** Quantification of results. Bar errors, SEM. ***P<0.001 (unpaired *t*-test). *ey>GFP* *Ras^V12^*cd *n*=44; *ey>GFP* *Ras^V12^*4DP *n*=51. **C** larval brains showing the invasion of GFP-labeled cells into the ventral nerve cord (VNC). Scale bar, 100 µm. **D** Quantification of VNC invasions. Bar errors, SEM (in at least three independent experiments). *** P<0.001 (chi square test). *ey>GFP* *Ras^V12^*cd *n*=44; *ey>GFP* *Ras^V12^*4DP *n*=51.

­

**
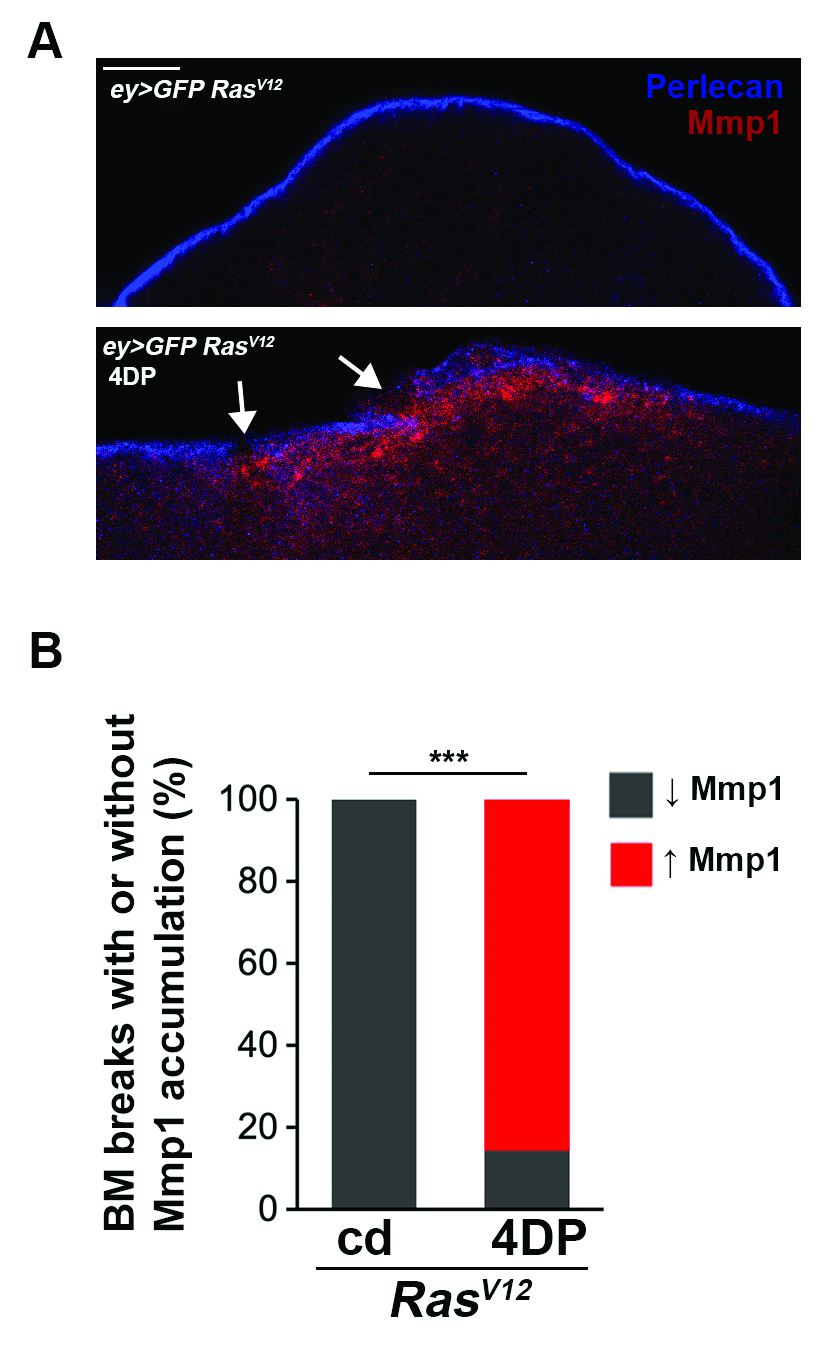
**

**Figure S3** Mmp1 accumulates in correspondence of BM breakages in *ey>GFP Ras^V12^* 4DP eye discs. A eye discs from *ey>GFP Ras^V12^* and *ey>GFP Ras^V12^* 4DP larvae stained for Perlecan and Mmp1. Upper panel shows an intact BM positive to the anti-Perlecan antibody which results associated to a low amount of Mmp1. The lower panel shows, instead, a less structured BM displaying gaps (arrowed), which is associated to Mmp1 accumulation. Scale Bar 20μm. **B** Quantification of results. ***p<0.001 (one sample *t*- test). *ey>GFP Ras^V12^* BM breaks *n*= 10; *ey>GFP Ras^V12^* 4DP BM breaks *n*= 21. BM=basement membrane; cd=control diet.


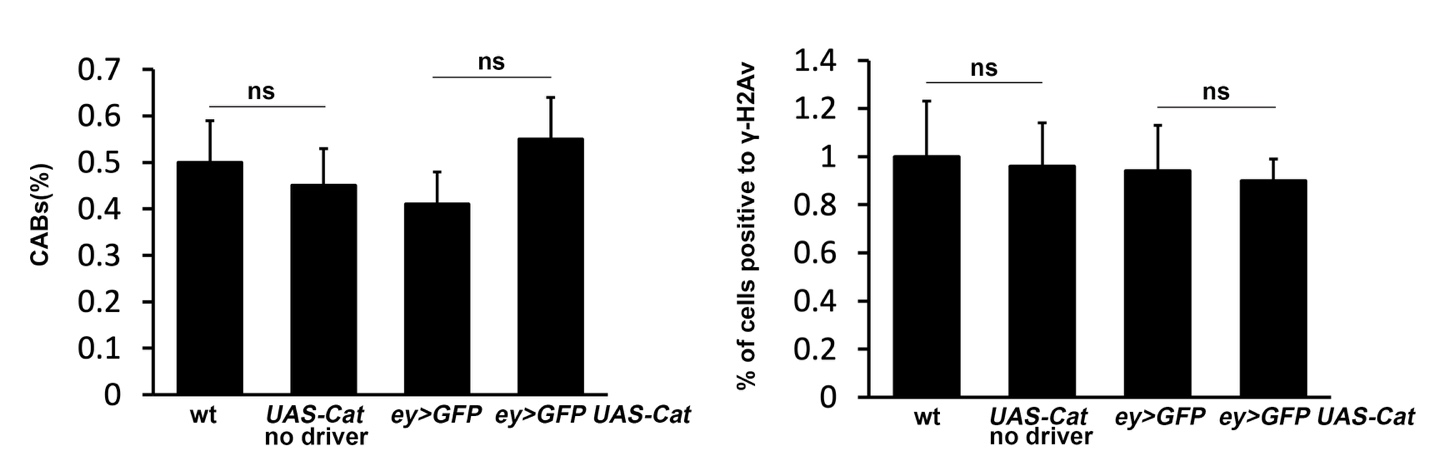


**Figure S4** Catalase overexpression do not increase CAB or DSB frequencies in control backgrounds. **A** CAB frequency in eye discs from wt larvae; larvae from the UAS-Cat line not crossed to driver; from *ey>GFP* and *ey>GFP UAS-Cat* larvae. wt=wild type; ns=not significant Error bars, SEM. *UAS-Cat* no driver vs wt P= 0.91; *ey>GFP UAS-Cat*; vs *ey>GFP* P=0.77 (chi square test). wt *n*= 400 (5 discs); *UAS-Cat* no driver *n=*444 (4 discs); *ey>GFP* *n*=480 (4 discs); *ey>GFP* *UAS-Cat* *n*=359 (4 discs). **B** Quantification of γ-H2Av positive nuclei. Error bars, SEM. *UAS-Cat* no driver vs wt P= 0.79; *ey>GFP UAS-Cat*; vs *ey>GFP* P=0.86 (chi square test). wt *n=*3002 (4 discs); *UAS-Cat* no driver *n*=2800 (3 discs); *ey>GFP* *n*=2027 (3 discs); *ey>GFP UAS-Cat* *n=*2021 (3 discs).


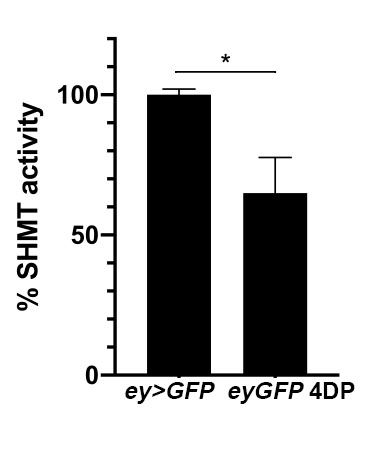


**Figure S5** Percentage of SHMT activity in eye discs from *ey>GFP* control larvae reared on control medium or on 4DP. 4DP treatment significantly decreases enzymatic activity. Error bars, SEM. *P<0.05 (unpaired *t*-test). *ey>GFP* *n=*100; *ey>GFP* 4DP *n=*100.


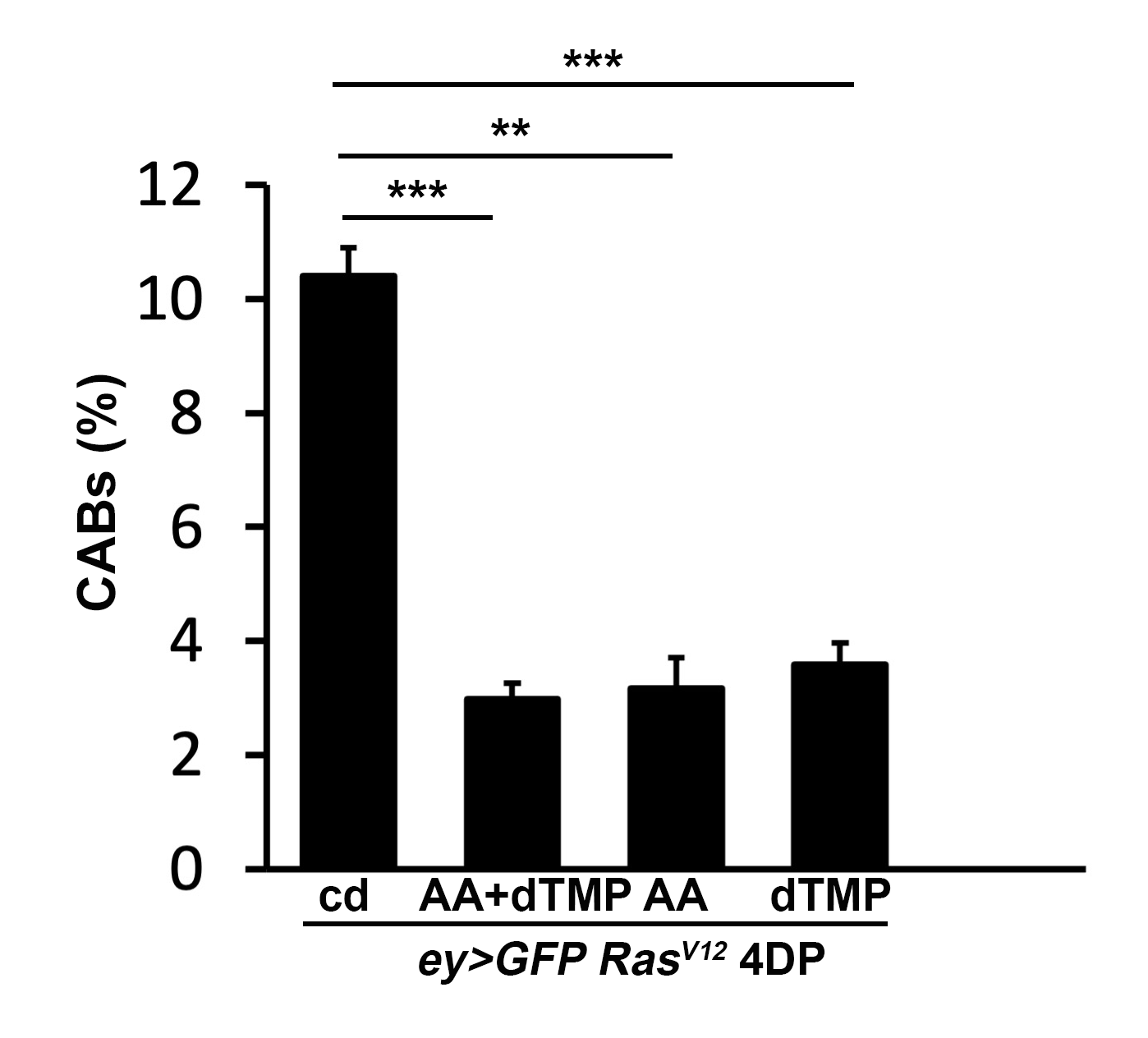


**Figure S6** In vitro treatment of eye discs from *ey>GFP* *Ras^V12^* 4DP-fed larvae with ascorbic acid (AA) and dTMP, alone or in combination, rescues CAB frequency. Error bars, SEM. **P<0.01, *** P<0.001 (chi square test) *ey>GFP* *Ras^V12^* 4DP (cd) *n*=259 (3 discs); *ey>GFP* *Ras^V12^* 4DP +AA+dTMP *n*=268 (3 discs); *ey>GFP* *Ras^V12^* 4DP+AA *n*=189 (5 discs); *ey>GFP* *Ras^V12^* 4DP+dTMP n=418 (4 discs).


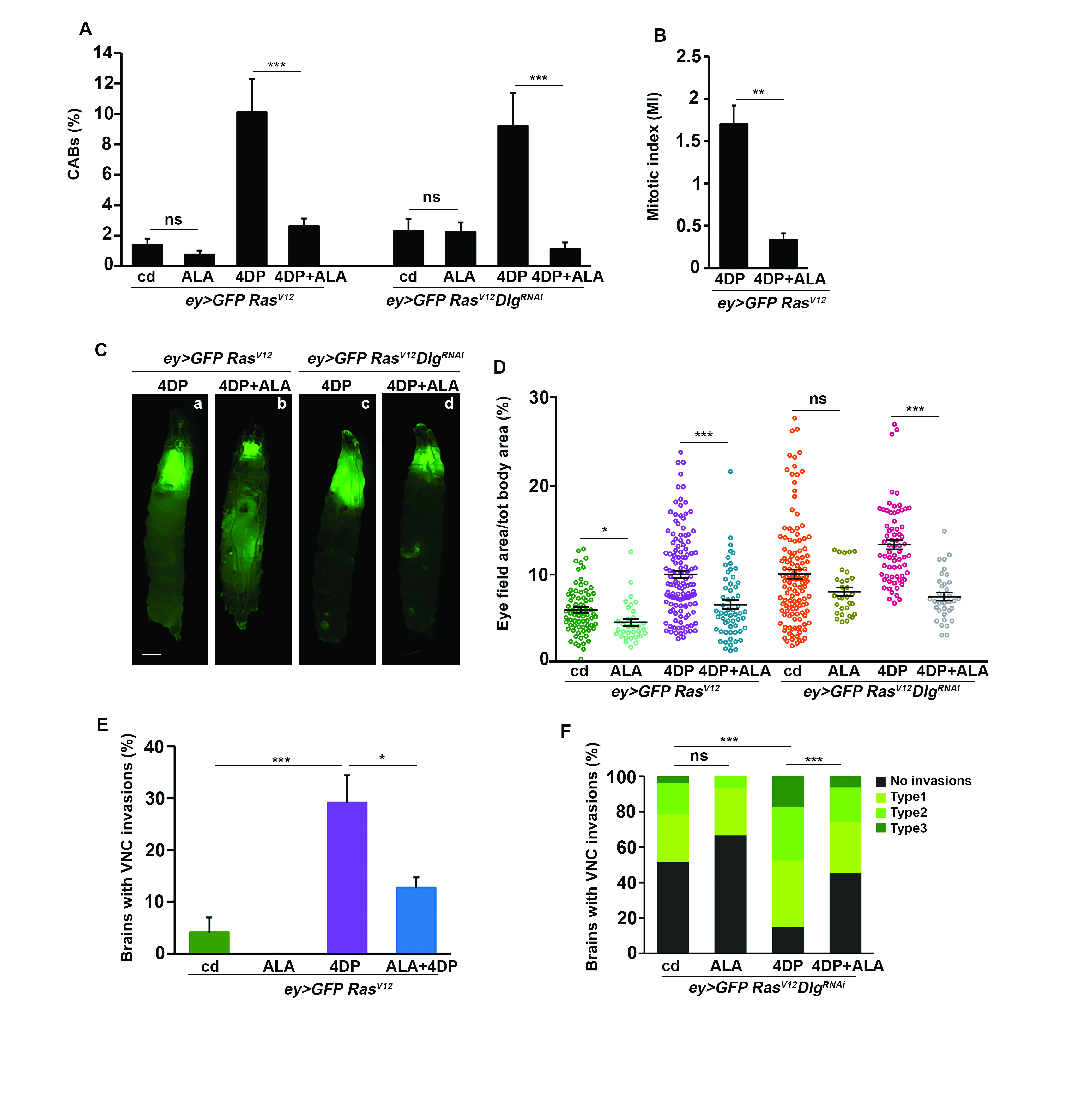
­**Figure S7** Alpha-lipoic acid (ALA) rescues CABs and tumor phenotypes **A** CAB frequency in *ey>GFP Ras^V12^* and *ey>GFP Ras^V12^Dlg^RNAi^* eye discs from larvae reared on cd, 4DP, ALA and 4DP+ALA. cd=control diet; 4DP=4-deoxypyridoxine; ALA= alpha-lipoic acid. Error bars, SEM. ***P<0.001 (chi square test). ns= not significant. *ey>GFP Ras^V12^*ALA vs *ey>GFP Ras^V12^*cd, P=0.4; *ey>GFP* *Ras^V12^Dlg^RNAi^*ALA vs *ey>GFP* *Ras^V12^Dlg^RNAi^* cd, P=0.92. Total number of examined cells in at least three independent experiments: *ey>GFP* *Ras^V12^* cd *n*=641 (11 discs); *ey>GFP* *Ras^V12^* ALA *n*=270 (6 discs); *ey>GFP* *Ras^V12^* 4DP *n*=642 (7 discs); *ey>GFP* *Ras^V12^* 4DP+ALA *n*=419 (13 discs); *ey>GFP* *Ras^V12^Dlg^RNAi^* cd *n=*339 (4 discs); *ey>GFP* *Ras^V12^Dlg^RNAi^*ALA *n*=268 ( 4 discs); *ey>GFP* *Ras^V12^Dlg^RNAi^* 4DP *n=*262 (5 discs); *ey>GFP* *Ras^V12^Dlg^RNAi^* 4DP+ALA *n=*710 (7 discs). **B** Mitotic index, MI (% of cells in mitosis) evaluated by anti-pH3 staining. Error bars, SEM. **P<0.01 (unpaired *t*-test). *ey>GFP* *Ras^V12^* 4DP *n*=19381 (3 discs); *ey>GFP* *Ras^V12^* 4DP+ALA *n*= 25217 (5 discs). **C** *ey>GFP Ras^V12^* and *ey>GFP Ras^V12^Dlg^RNAi^* larvae reared on 4DP (a,c) or 4DP+ALA (b,d). Scale bar, 0.5mm. **D** Quantification of GFP-positive eye field area relative to total body area. Error bars, SEM. *P<0.05, ***P<0.001 (unpaired *t*-test). ns=not significant (P=0.066). *ey>GFP* *Ras^V12^* cd *n*=73; *ey>GFP* *Ras^V12^* ALA *n*=32; *ey>GFP* *Ras^V12^*4DP *n*=129; *ey>GFP* *Ras^V12^* 4DP+ALA *n*=58 ; *ey>GFP* *Ras^V12^ Dlg^RNAi^* cd *n*=126; *ey>GFP* *Ras^V12^ Dlg^RNAi^* ALA *n*=31; *ey>GFP* *Ras^V12^ Dlg^RNAi^* 4DP *n*=69; *ey>GFP* *Ras^V12^ Dlg^RNAi^* 4DP+ALA *n*=35. **E** Quantification of VNC invasions in *ey>GFP* *Ras^V12^* larvae. Error bars, SEM. *P<0.05, ***P<0.001 (chi square test). *ey>GFP* *Ras^V12^*cd *n*=48; *ey>GFP* *Ras^V12^* ALA *n*=33; *ey>GFP* *Ras^V12^*4DP *n*=55; *ey>GFP* 4DP+ALA *n*=55. **F** Quantification of VNC invasions in *ey>GFP* *Ras^V12^Dlg^RNAi^* larvae. The green-labeled portion of each column represents the percentage of brains with VNC invasions. The black portion the percentage of brains without invasions. The three different types of green represent arbitrary levels of invasion. Type1=mild phenotype; type2=moderate phenotype; type 3=severe phenotype (for examples see Fig. 3C or Fig. 4G). Quantification of results. Statistics was assessed by chi square test and refers to the percentage of invasion phenotype. *** P<0.001. ns=not significant (P=0.1509). *ey>GFP* *Ras^V12^Dlg^RNAi^ n*=70; *ey>GFP* *Ras^V12^Dlg^RNAi^*+ALA *n*=30; *ey>GFP* *Ras^V12^Dlg^RNAi^* 4DP *n*=80 *ey>GFP* *Ras^V12^Dlg^RNAi^* 4DP+ALA *n*=31.
